# A signature of plasma exosomal miRNAs predict therapeutic efficacy to neoadjuvant immunotherapy in patients with non-small cell lung cancer

**DOI:** 10.1515/jtim-2025-0052

**Published:** 2025-12-12

**Authors:** Wenyu Zhai, Yaobin Lin, Yuheng Zhou, Qinglin Wang, Zerui Zhao, Shoucheng Feng, Bingyu Rao, Weizhen Sun, Yizhi Wang, Zhigang Zhou, Jing Qin, Hao Long

**Affiliations:** State Key Laboratory of Oncology in South China, Guangdong Provincial Clinical Research Center for Cancer, Sun Yat-Sen University Cancer Center, Guangzhou, Guangdong Province, China; Lung Cancer Research Center, Sun Yat-Sen University, Guangzhou, Guangdong Province, China; Department of Thoracic Surgery, Zhujiang Hospital, Guangzhou, Guangdong Province, China; Department of Immunology and Oncode Institute, Leiden University Medical Center, Leiden, The Netherlands; Changde Hospital, Xiangya School of Medicine, Central South University (The first people's hospital of Changde city), Changde, Hunan Province, China

## To the editor

Lung cancer remains a leading cause of cancer-related deaths globally,^[1]^ with non-small cell lung cancer (NSCLC) being the most prevalent subtype.^[2]^ Over 35% of NSCLC cases are diagnosed at a locally advanced stage, where neoadjuvant immune checkpoint inhibitor (ICI) therapy has a standard strategy for resectable patients.^[3]^ Consequently, identifying robust biomarkers to optimize candidate selection and therapeutic strategies is crucial.^[4]^

Exosomes are nano-sized vesicles secreted by various cells, including cancer cells, and facilitate intercellular communication *via* proteins, lipids, and RNAs.^[5]^ They play a key role in modulating the tumour microenvironment and immune responses to therapy.^[6]^

MicroRNAs (miRNAs) are key gene expression regulators.^[7]^ Exosomal miRNAs significantly influence the immune landscape; tumour-derived ones can suppress immune cell function, promoting immune evasion. For instance, colorectal cancer-derived hsa-miR-21-5p and hsa-miR-200a synergistically induce macrophage M2-like polarisation and upregulate programmed cell death-ligand 1 (PD-L1) expression, thereby suppressing CD8^+^ T-cell activity and promoting tumour growth.^[8]^ Besides, exosomal miRNAs offer superior clinical utility. Their stability in circulation and compatibility with high-sensitivity, low-cost reverse transcription quantitative real-time PCR (RT-qPCR) make them ideal for liquid biopsies, unlike proteins or lipids requiring complex proteomic/Lipidomic workflows. Emerging evidence specifically implicates plasma exosomal miRNAs in predicting NSCLC immunotherapy outcomes, whereas other exosomal cargoes lack comparable validation. These make exosomal miRNAs have the potential to become predictive biomarkers for neoadjuvant immunotherapy responses.^[9]^

This study analysed 40 plasma samples (pre- and post-treatment) from 20 NSCLC patients (10 pCR, 10 non-pCR) in a clinical trial (NCT05244837),^[10]^ plus five healthy volunteer samples. Exosomal miRNA sequencing identified expression changes related to neoadjuvant immunotherapy. A retrospective cohort of 48 pre-treatment samples was used to develop a pCR-predictive miRNA signature.

### Patient characteristics

The study included a prospective discovery cohort (*n* = 20) and a retrospective training cohort (*n* = 48) receiving neoadjuvant immunochemotherapy (Supplementary Figure S1A). The details of patient characteristics were shown in Supplementary Table S1. The median follow-up duration for the training cohort was 26.6 months.

### Exosmoal miRNA-seq in the discovery cohort

**Figure 1:**
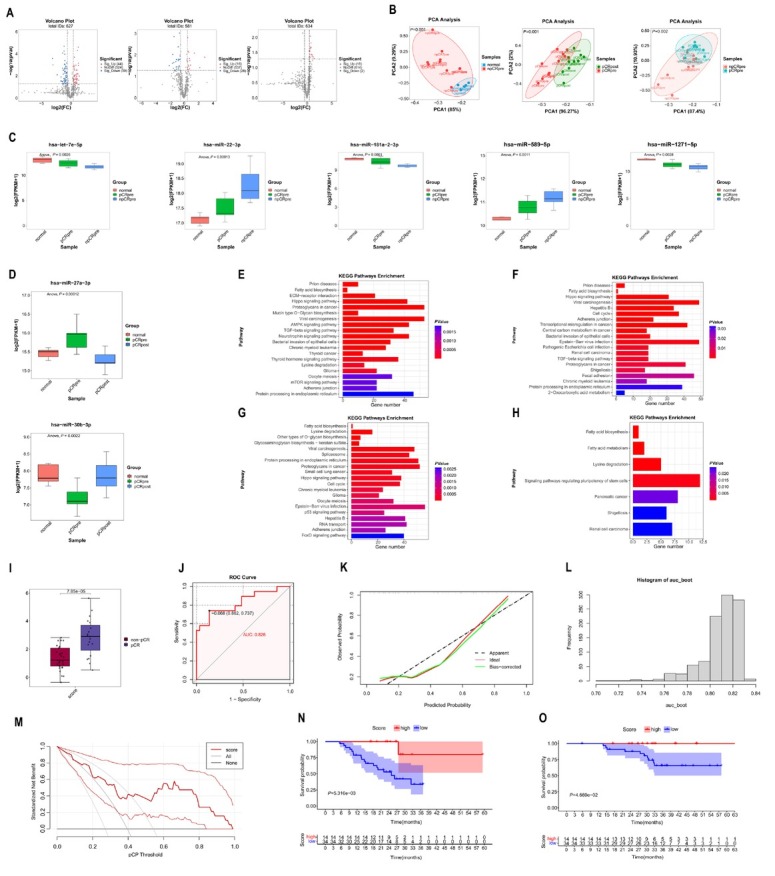
Differential expression, KEGG analyses of miRNAs and construction of the 5-miRNA signature for predicting pCR in discovery cohort. (A) Volcano plot showing the differentially DEmiRNAs in pre-treatment *versus* healthy group (left), pre-treatment *versus* post-treatment group (middle), and pCR *versus* non-pCR group (right). (B) PCA plot showing that DEmiRNAs could clearly stratify samples into non-pCR *versus* healthy group (left), pre-treatment *versus* post-treatment group (middle), and pCR *versus* non-pCR group (right). (C) Boxplot showing that let-7e-5p, miR-181a-2-3p, and miR-1271-5p up-regulated in healthy and pCR samples, while miR-22-3p and miR-589-5p down-regulated in healthy and pCR samples. (D) Boxplot showing that miR-27a-3p up-regulated in pre-treatment samples and down-regulated after neoadjuvant therapy, while miR-30b-3p down-regulated in pre-treatment samples and up-regulated after neoadjuvant therapy. (E) The top 20 pathways in KEGG analyses of 3 miRNAs which up-regulated in healthy and pCR samples. (F) The top 20 pathways in KEGG analyses of 2 miRNAs which down-regulated in healthy and pCR samples. (G) The top 20 pathways in KEGG analyses of miR-27a-3p. (H) The 7 pathways in KEGG analyses of miR-30b-3p. (I) Boxplot showing the significantly higher score in pCR patients than in the non-pCR patients. (J) The receiver operating characteristic curve of pCR score. (K) The calibration curve of pCR score. Predicted and actual pCR probability were respectively plotted on the X-axis and the Y-axis. The 45-degree dashed lines through the coordinate origin represent the excellent calibration models. (L) The distribution of AUC for 1000 times bootstrapping. (M) The DCA of the clinical value for the pCR score. (N) Event-free survival for patients with pCR and non-pCR. (O) Overall survival for patients with pCR and non-pCR. KEGG: Kyoto Encyclopedia of Genes and Genomes; PCA: principal component analysis; DEmiRNAs: differential expression miRNAs; PCA: principal component analysis; AUC: areas under the ROC; DCA: decision curve analysis.

Exosomes were isolated using ultracentrifugation and characterised by transmission electron microscopy, particle size analysis, and western blotting for specific markers, including TSG101 and CD81 (Supplementary Figure S1B). Total RNA was extracted from the purified exosomes, and miRNA sequencing was performed on samples from the discovery cohort and healthy volunteers. Differentially expressed miRNAs (DEmiRNAs) were analysed across three comparisons: pre-treatment *versus* healthy samples, pre-treatment *versus* post-treatment samples in pCR patients, and pre-treatment samples between pCR and non-pCR patients. 103 DEmiRNAs (*P* < 0.05, fold change > 1.4) were identified, with 44 miRNAs upregulated and 59 downregulated in pre-treatment compared with healthy samples (Figure 1A). The top five upregulated and downregulated DEmiRNAs are listed in Supplementary Table S2. Forty-four DEmiRNAs were identified, including 16 upregulated and 28 downregulated in pre-treatment compared with post-treatment samples (Figure 1A). The top five upregulated and downregulated miRNAs are presented in Supplementary Table S3. Twenty DEmiRNAs were identified, with 18 upregulated and 2 downregulated in pCR compared with non-pCR samples (Figure 1A). The top five upregulated and two downregulated DEmiRNAs are shown in Supplementary Table S4. Principal component analysis (PCA) revealed that DEmiRNAs effectively distinguished pre-treatment from post-treatment samples and pCR from non-pCR samples (Figure 1B). Interestingly, PCA could differentiate healthy from pre-treatment non-pCR samples but not from pCR samples (Figure 1B; Supplementary Figure S1C). A heatmap of DEmiRNA expression profiles is shown in Supplementary Figure S1D. The intersection of DEmiRNAs between healthy *versus* pre-treatment and pCR *versus* non-pCR samples identified let-7e-5p, miR-181a-2-3p, and miR-1271-5p were upregulated in healthy and pCR samples, while miR-22-3p and miR-589-5p were downregulated in both groups (Figure 1C). The intersection between healthy *versus* pre-treatment samples and pre-treatment *versus* post-treatment samples revealed miR-27a-3p, which was upregulated in pre-treatment samples and downregulated post-treatment, and miR-30b-3p, which was downregulated in pre-treatment samples and upregulated post-treatment (Figure 1D). Kyoto Encyclopedia of Genes and Genomes (KEGG) pathway analysis was conducted to identify the potential functions of the identified DEmiRNAs. The top 20 enriched pathways for the three miRNAs upregulated in healthy samples, two miRNAs upregulated in non-pCR samples, and miR-27a-3p are displayed as bar plots (Figures 1E-G). Seven enriched pathways for miR-30b-3p are shown in Figure 1H. Notably, the fatty acid biosynthesis pathway emerged as a common enriched pathway across the four KEGG analyses, highlighting a metabolic axis where tumour cells, immune cells, and exosomal miRNAs intersect to shape immunotherapy responses. Mechanistically, this pathway may drive resistance by fostering an immunosuppressive lipid-rich tumour microenvironment or sensitize tumour *via* ferroptosis susceptibility. Further experimental exploration is needed to clarify these interactions. If validated, targeting fatty acid biosynthesis could emerge as a strategy to augment neoadjuvant immunotherapy in NSCLC.

### Construction of 5 exosomal miRNA signatures for predicting pCR and prognostic stratification

The expression levels of five miRNAs (let-7e-5p, miR-181a-2-3p, miR-1271-5p, miR-22-3p and miR-589-5p) were measured using RT-qPCR in the training cohort. Least absolute shrinkage and selection operator (LASSO) regression selected all five miRNAs with the optimal log lambda value of -5.830026 (Supplementary Figure S1E). A pCR signature, named as “pCR score”, was constructed. The specific formula for the pCR score = miR-1271-5p*0.879 + let-7e-5p*0.490 + miR-181a-2-3p*0.888 – miR-589-5p*1.308 – miR-22-3p*0.358. As shown in Figure 1I, pCR patients exhibited significantly higher pCR scores. With an area under the receiver operating characteristic curve (AUC) of 0.826, the pCR score demonstrated satisfactory predictive performance (Figure 1J). The calibration curve demonstrated good agreement between the predicted and actual probabilities of pCR (Figure 1K). Internal bootstrap validation, performed with 1000 repetitions, yielded a median AUC of 0.813 (95% confidence interval [CI]: 0.764–0.829; Figure 1L). Additionally, decision curve analysis (DCA) indicated that using the pCR score for clinical decision-making provides a net benefit for patients undergoing neoadjuvant immunotherapy (Figure 1M). Based on a cut-off value of 2.60, derived from maximally selected log-rank statistics (Supplementary Figure S1F), patients were stratified into two groups: high pCR score (14 patients) and low pCR score (34 patients). Among patients with a high pCR score, only one experienced event-free survival (EFS) event and no patients had overall survival (OS) event, while 18 patients with a low pCR score experienced EFS events and 14 patients experienced OS events. As shown in Figure 1N, patients with a high pCR score had significantly longer EFS compared to those with a low pCR score (log-rank test *P*= 0.005). Similarly, patients with a high pCR score also had better OS than those with a low pCR score (log-rank test *P* = 0.047, Figure 1O). Though, the median follow-up duration of 26.6 months is shorter than the median EFS (31.6 months) in checkmate 816 trial, historical precedents suggest that early survival advantages in biomarker-stratified NSCLC cohorts often persist, particularly when linked to pathological response. Therefore, the pCR score represents a promising but preliminary tool for risk stratification. However, late recurrences may modestly alter survival curve and we would concentrate prolonged follow-up outcomes.

This study highlights exosomal miRNAs as promising biomarkers for predicting pCR in NSCLC patients receiving neoadjuvant immunotherapy. We identified significant changes in the expression of two miRNAs (miR-27a-3p and miR-30b-3p) following neoadjuvant immunotherapy. Furthermore, five miRNAs (let-7e-5p, miR-181a-2-3p, miR-1271-5p, miR-22-3p and miR-589-5p) were found to correlate strongly with pCR outcomes. The developed model for predicting pCR shows high accuracy and reliability, offering a valuable tool for patient selection and personalized treatment.

## Supplementary Information

Supplementary materials are only available at the official site of the journal (www.intern-med.com).

## Supplementary Material

Supplementary Material Details
