# Supplementary material for: A signature of plasma exosomal miRNAs predict therapeutic efficacy to neoadjuvant immunotherapy in patients with non-small cell lung cancer
Source: J Transl Int Med. 2025 Dec 12;13(6):614–7. doi: 10.1515/jtim-2025-0052 (PMC12721357; doi:10.1515/jtim-2025-0052)
Supplement: Supplementary file 1 — Supplementary Material Details [file jtim-2025-0052_sm.pdf]

MATERIALS AND METHODS

Study design and patient population

This is a multi-cohort study with a discovery cohort and a training cohort. The discovery cohort included 20 non-small cell lung cancer (NSCLC) patients from the LungMark study (NCT05244837). LungMark study is a prospective Phase II clinical trial designed to evaluate the efficacy and safety of Tiragolumab in combination with platinum-based doublet chemotherapy for the treatment of NSCLC. For patients who are resectable, an initial treatment regimen consisting of three cycles of Tislelizumab combined with platinum-based doublet chemotherapy will be administered, followed by surgical resection. Ten pairs of patients with pathologic complete response (pCR) and non-pathologic complete response (non-pCR) after neoadjuvant immunotherapy and five healthy volunteers who served as healthy controls were collected as discovery cohort. 48 NSCLC patients who underwent neoadjuvant immunotherapy at Sun Yat-sen University Cancer Center between October 2019 and September 2022 were retrieved as training cohort. Exosome miRNA sequencing was conducted on plasma samples from the discovery to identify potential predictive biomarkers. The 48 retrospective plasma samples will be utilized to develop a predictive model for pCR following neoadjuvant immunotherapy. Based on the multidisciplinary recommendations from the International Association for Lung Cancer Research, pCR is defined as the presence of no residual cancer cells within the primary tumor bed and lymph node.

Isolation and identification of exosomes.

Incubate the sample at 37 ℃ with moderate agitation. Transfer the sample to a new centrifuge tube and centrifuge (Microfuge 20R, Beckman, USA) at 2000 × g, 4 ℃ for 30 min. Carefully transfer the supernatant to a new tube and centrifuge at 10,000 × g, 4 ℃ for 45 min to remove larger vesicles. Filter the supernatant through a 0.45 μm filter membrane and collect the filtrate. Transfer the filtrate to a new tube and centrifuge using a high-speed rotor at 100,000 × g, 4 ℃ for 70 min. Discard the supernatant, resuspend the pellet in 10 mL of pre-chilled 1× PBS (Sangon Biotech, China), and centrifuge again at 100,000 (CP100MX, Hitachi, Japan) × g, 4 ℃ for 70 min. Discard the supernatant and resuspend the pellet in 100 μL of pre-chilled 1× PBS and take 10 μL for transmission

electron microscopy (TEM) observation, and 10 μL for particle size analysis.

10 μL of the sample was pipetted onto a copper grid and allowed to settle for 1 min, followed by removal of excess liquid using filter paper. Then, 10 μL of uranyl acetate (GZ02625, EMCN, China) was added to the copper grid and allowed to settle for 1 min, after which excess liquid was removed using filter paper. The sample was air-dried for several minutes at room temperature before imaging using TEM (HT-7700, Hitachi, Japan) at 100 kV.

Take 10 μL of the exosome sample and dilute it to 30 μL. Perform an instrument performance test using a standard sample, and proceed with exosome sample injection into the particle size analyzer (N30E, NanoFCM, China) only if the instrument passes the test. Note that gradient dilution should be performed to prevent clogging of the injection needle.

We denatured the exosome supernatant in 5 × sodium dodecyl sulfonate (SDS) buffer (10% SDS-polyacrylamide gel electrophoresis; 50 μg protein/Lane) and subjected it to western blot analysis. Rabbit polyclonal antibodies against CD81 (Abcam, USA) and TSG101 (Abcam, USA) were used in western blot analysis.

Exosomal RNA isolation and analyses

According to the kit instructions, resuspend the exosome pellet in 100–200 μL ultrapure water, thaw, and mix thoroughly. Add 1 mL of Trizol (Thermo, USA) to the exosome suspension, mix well, and incubate on ice for 30 min. Add 200 μL chloroform, mix well, and incubate on ice until the solution separates into distinct layers. Centrifuge at 4 ℃, 12,000 rpm for 5 min and collect the supernatant. Follow the protocol for further purification using isopropanol and ethanol. Measure 2 μL of RNA using a NanoDrop 2000 (Thermo, USA) to assess RNA concentration and the A260/A280 ratio.

Real-time quantitative PCR (RT-qPCR)

Use a commercial reverse transcription kit (TUREscript 1st Stand cDNA SYNTHESIS Kit, Aidlab, China) to synthesize cDNA, following the manufacturer's instructions. Details for primers of U6 (reference genes), let-7e-5p, miR-181a-2-3p, miR-1271-5p, miR-22-3p, miR-589-5p, can be found in the following table. Set up the PCR reaction and run the cycle as per the standard RT-qPCR protocol. Record the Ct values for analysis.

primers	primer sequence (5'—3')
U6-F (reference genes)	CTCGCTTCGGCAGCACATATACT
U6-R (reference genes)	ACGCTTCACGAATTTGCGTGTC
hsa-miR-22-3p-F	GGAAGCTGCCAGTTGAAG
hsa-miR-22-3p-R	GCAGGGTCCGAGGTATTC
hsa-let-7e-5p-F	GGTGAGGTAGGAGGTTG
hsa-let-7e-5p-R	GCAGGGTCCGAGGTATTC
miR-1271-5p-F	GATCGCTTGGCACCTAG
miR-1271-5p-R	GCAGGGTCCGAGGTATTC
miR-181a-2-3-F	GCACCACTGACCGTTGAC
miR-181a-2-3p-R	GCAGGGTCCGAGGTATTC
miR-589-5p-F	GTTTCAGGCTCAGGTCAG
miR-589-5p-R	GCAGGGTCCGAGGTATTC

miRNA library construction and sequencing

3' adapter ligation

In an ice bath, mix 1 μg total RNA with 1 μL of RNA 3' Adapter. Incubate at 70 ℃ for 2 min, then place on ice. Add 2 μL Ligation Buffer, 1 μL RNase Inhibitor, and 1 μL T4 RNA Ligase 2, mix well, and incubate at 28 ℃ for 1 h. Add 1 μL Stop Solution, mix, and incubate for another 15 min.

5' adapter ligation

Mix 50 μL of RNA 5' Adapter with 200 μL in the PCR tube. Incubate at 70 ℃ for 2 min, then place on ice. Add ATP, T4 RNA Ligase, and other reagents as per the protocol, and incubate at 28 ℃ for 1 h.

RNA reverse transcription

Mix dNTPs, adapters, and RNA RT Primer. Incubate at 70 ℃ for 2 min, then place on ice. Add SuperScript II Reverse Transcriptase and incubate at 50 ℃ for 1 h.

RNA amplification and purification

Amplify the cDNA using a PCR protocol. Purify the final library using electrophoresis and gel extraction.

Sequencing

Sequence the library on an Illumina HiSeq 2500 with a 1x50bp single-end read.

Expression data preprocessing

Use Feature Extraction (version 12.0.3.1, Agilent, USA) to process raw images and extract data. Normalize the data using GeneSpring (version 14.8, Agilent, USA) and filter probes that were detected in at least 80% of the samples in the comparison group. Filter raw sequencing data to remove adapter reads and low-quality sequences. Align the cleaned reads against miRNA databases to filter out non-miRNA data. The processing of raw data and miRNA mapping were achieved using the ACGT101-miR (LC Sciences, Houston, Texas, USA) and miRBase 22.0 (http://www.mirbase.org/). And miRNA expression data were normalized for FPKM by with edgeR software.

Statistical analyses

We carried out the differential expression analysis *via* t-test to identify the differentially expressed miRNAs (DEmiRNAs). A miRNA with |fold change| > 1.4 and *P* value < 0.05 was defined as DEmiRNA. Analysis of Variance was used to compare the expression level of miRNAs among different groups of samples. We executed the Kyoto Encyclopedia of Genes and Genomes (KEGG) enrichment analysis of selected DEmiRNA through the DIANA Tools (diana.e-ce.uth.gr/home). The qPCR data were normalized based on the U6 genes, and the 2-ΔΔCT values were calculated as the relative quantification. *P* values for the significance of differences between the qPCR relative quantification of pCR and non-pCR samples were calculated using the Wilcoxon method. The least absolute shrinkage and selection operator (LASSO) algorithm was used to select the miRNAs for predicting pCR and calculating their corresponding coefficients. LASSO can compress the regression coefficients of some unnecessary variables to zero and then remove them from the model to achieve the purpose of variable screening, ensuring the simplicity and stability of the model. And it also realizes the combination and optimization of the characteristics of the optimal subset and ridge regression. When applied to variable screening, LASSO can be used no matter it is continuous, binary, or multi-classification; it is effective in processing high-dimensional small sample data and solving multicollinearity problems. A pCR signature was constructed, and its predictive score (pCR score) was summed up using the relative quantification of selected miRNAs and their corresponding regression coefficients.

The specific formula was as follows: pCR score = sum (selected miRNAs × corresponding coefficients). The predictive performance of this pCR score was evaluated by calculating the area under the curve (AUC) of the receiver operating characteristic (ROC) and calibration curves. Furthermore, we performed 1000 times bootstrap resampling to validate its predictive capability. In addition, the clinical value of this pCR score was assessed by decision curve analysis (DCA). The maximally selected log-rank test was used *via* the "maxstat" R package to determine the cut-off value of pCR score for converting pCR score into a binary categorical variable. Survival curves for event-free survival were estimated using the Kaplan-Meier method and compared through the log-rank test. Statistical analyses in this study were performed using R software (version 4.4.1, Vanderbilt University, Nashville, TN, USA). A two-tailed P value < 0.05 was considered statistically significant.

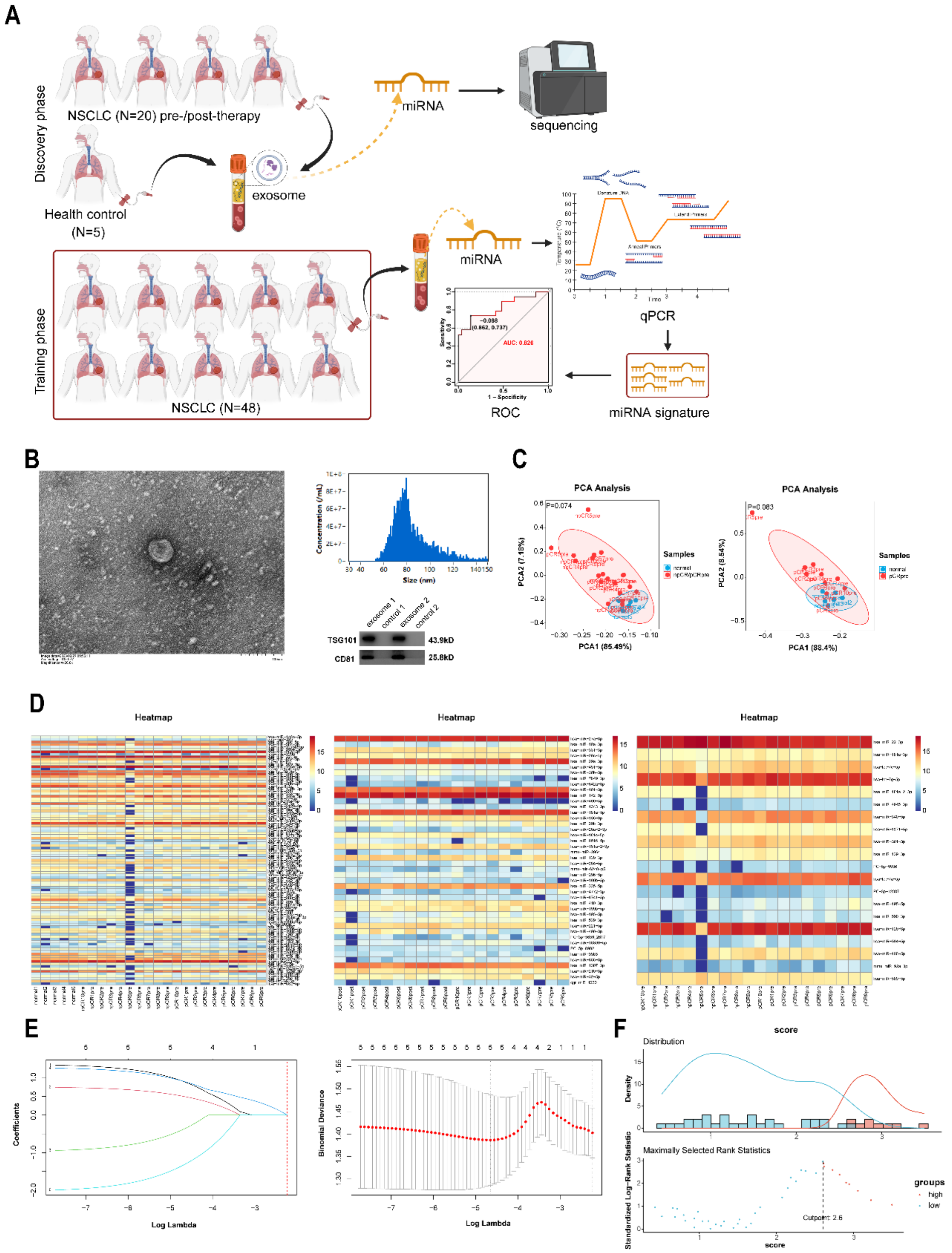

Supplemental Figure S1: (A) The flow chart of this study. (**B)** Representative electron microscope image of purified plasma-derived exosome (left): Representative particle size analysis plot of purified plasma-derived exosome (upper right): western blot showing expression of CD81 and TSG101 in purified plasma-derived exosome (lower right). (**C)** PCA plot showing that DEmiRNAs could not clearly stratify samples into pre-treatment versus healthy group (left) and pCR versus healthy group (left). (**D)** The heatmap showing

the expression of DEmiRNAs in pre-treatment versus healthy group (left): pre-treatment versus post-treatment group (middle): and pCR versus non-pCR group (right). (**E)** LASSO coefficients of miRNAs: each curve represents a miRNA (left): selection of the optimal candidate miRNAs in the LASSO model (right). (**F)** Determination of cut-off value of pCR score by the maximally selected log-rank statistics. PCA: principal component analysis; LASSO: least absolute shrinkage and selection operator.

Supplemental Table S1: The baseline characteristics of patients in discovery and training cohort

**Characteristics**	**Average or case No. (%) discovery cohort (*n* = 20)**	**Average or case No. (%) training cohort (*n* = 48)**	**Average or case No. (%) healthy volunteer (*n* = 5)**
Gender			
Male	18 (90.0)	43 (89.6)	3 (60.0)
Female	2 (10.0)	5 (10.4)	2 (40.0)
Age (year)	62.6±4.2	59.4±9.0	33.0±2.2
Smoking history			
No	3 (15.0)	11 (22.9)	4 (80.0)
Yes, or ever	17 (85.0)	37 (77.1)	1 (20.0)
cT stage[a]			
T1	3 (15.0)	3 (6.3)	
T2	7 (35.0)	16 (33.3)	
T3	6 (30.0)	13 (27.1)	
T4	4 (20.0)	16 (13.3)	
cN stage[a]			
N0	1 (5.0)	4 (8.3)	
N1	2 (10.0)	10 (20.8)	
N2	14 (70.0)	21 (53.8)	
N3	3 (15.0)	13 (27.1)	
Differentiation degree			

Moderate	6 (30.0)	12 (25.0)
Poor	11 (55.0)	34 (70.8)
Undifferentiation	3 (15.0)	2 (4.2)
Histological type		
LUSC	18 (90.0)	29 (60.4)
LUAD	2 (10.0)	12 (25.0)
Others[b]	0 (0)	7 (14.6)
pCR		
Yes	10 (50.0)	19 (39.6)
No	10 (50.0)	29 (60.4)

[a] Diagnosed based on the AJCC criteria (8th edition). [b] Including lung lymphoepithelioma-like carcinoma, carcinoid. LUSC: Lung Squamous Cell Carcinoma; LUAD: lung adenocarcinoma.

Supplemental Table S2: The top 5 up-regulated and down-regulated DEmiRNAs in pre-treatment versus healthy samples

**miR_name**	**miR_seq**	**log2 FC**	***P* value**
hsa-miR-148a-5p	AAAGTTCTGAGACACTCCGACT	1.04	2.00E-05
hsa-miR-584-5p	TTATGGTTTGCCTGGGACTGA	1.05	5.59E-05
hsa-miR-1273h-5p	CTGGGAGGTCAAGGCTGCAGTGT	1.28	8.45E-05
hsa-miR-548e-3p	AAAAACTGAGACTACTTTTGC	0.92	1.05E-04
hsa-miR-1290	TGGATTTTTGGAGCAGGGA	2.98	2.54E-04
oga-miR-28b	TCGAGGAGCTCACAGTCTAGTT	-1.32	2.43E-05
hsa-miR-30c-5p	TGTAAACATCCTACACTCTCAGCT	-1.19	4.73E-05
hsa-miR-26a-5p	TTCAAGTAATCCAGGATAGGCT	-0.67	1.99E-04
bta-miR-150	TCTCCCAACCCTTGTACCAGTGT	-1.35	6.88E-04
hsa-let-7a-5p	TGAGGTAGTAGGTTGTATAGTT	-0.94	1.07E-03

DEmiRNAs, differentially expressed miRNAs.

Supplemental Table S3: The top 5 up-regulated and down-regulated DEmiRNAs in pre-treatment versus pre-operation samples

**miR_name**	**miR_seq**	**log2 FC**	***P* value**
hsa-miR-331-3p	CCCCTGGGCCTATCCTAGAAT	0.52	1.49E-03
hsa-miR-491-5p	AGTGGGGAACCCTTCCATGAGG	0.57	6.53E-03
hsa-miR-30b-3p	CTGGGAGGTGGATGTTTACTTC	0.67	7.21E-03
hsa-miR-7849-3p	GACAATTGTTGATCTTGGGCCT	1.44	7.89E-03
hsa-miR-490-5p	CCATGGATCTCCAGGTGGGT	2.88	1.24E-02
hsa-miR-27a-3p	TTCACAGTGGCTAAGTTCCG	-0.62	9.21E-04
hsa-miR-10a-3p	CAAATTCGTATCTAGGGGAAT	-1.37	1.34E-03
hsa-miR-29c-3p	TAGCACCATTTGAAATCGGTT	-0.78	4.00E-03
hsa-miR-29a-3p	TAGCACCATCTGAAATCGGTT	-0.82	4.05E-03
hsa-miR-642a-3p	AGACACATTTGGAGAGGGAAC	-1.54	9.02E-03

DEmiRNAs: differentially expressed miRNAs.

Supplemental Table S4: The top 5 up-regulated and 2 down-regulated DEmiRNAs in pCR versus non-pCR samples

**miR_name**	**miR_seq**	**log2 FC**	***P* value**
hsa-miR-181a-3p	ACCATCGACCGTTGATTGTACC	0.67	8.85E-03
hsa-let-7e-5p	TGAGGTAGGAGGTTGTATAGTT	0.83	1.49E-02
hsa-let-7g-5p	TGAGGTAGTAGTTTGTACAGTT	0.58	1.98E-02
hsa-miR-181a-2-3p	ACCACTGACCGTTGACTGTAC	0.79	2.10E-02
hsa-miR-4645-3p	CGAGACAGTAGTTCTTGCCTGGT	0.84	2.10E-02
hsa-miR-22-3p	AAGCTGCCAGTTGAAGAACTGT	-0.85	7.00E-03
hsa-miR-589-5p	TGAGAACCACGTCTGCTCTGA	-0.75	4.93E-02

DEmiRNAs, differentially expressed miRNAs.
